# Unlocking health equity by eliminating copayments for essential antihypertensive medications

**DOI:** 10.1016/j.eclinm.2025.103094

**Published:** 2025-01-30

**Authors:** Thomas R. Frieden, Renu Garg, Bolanle Banigbe, Sohel Choudhury, Nanlop Ogbureke, Dereje Duguma, Viroj Tangcharoensathien

**Affiliations:** a Resolve to Save Lives, New York, NY, USA; b National Heart Foundation, Dhaka, Bangladesh; c Resolve to Save Lives, Abuja, Nigeria; d Ministry of Health, Addis Ababa, Ethiopia; e International Health Policy Program, Nonthaburi, Thailand

**Keywords:** Hypertension, Antihypertensive medications, Medication adherence, Medication cost, Health care financing

## Abstract

Hypertension kills more people than any other condition; approximately 80% of deaths occur in low- and middle-income countries (LMICs). Only about 1 in 5 adults with hypertension worldwide and less than 1 in 10 in LMICs have their condition controlled. Adherence to antihypertensive medications increases control and decreases hospitalizations, strokes, heart attacks, and health care costs. Eliminating patient copayments for antihypertensive medications increases adherence and hypertension control. This powerful but underutilized strategy can advance universal health coverage and reduce economic hardship. Medication access with no out-of-pocket cost to patients is feasible and economically sound, but requires increased investment and careful implementation to avoid unintended consequences of reducing flexible funding needed for primary health care systems. Provision of medications that are free to patients at the point of care should be part of a multi-component, sustainable approach to addresses systemic barriers to treatment of hypertension, the world's leading cause of death.

**Search strategy and selection criteria**

References for this article were identified through searches of PubMed with the search terms “free,” “no co-pay,” and “no cost”; “drugs,” “medicines,” and “medications”; and “blood pressure,” “hypertension,” and “noncommunicable disease.” We did not limit our search to a specific timeframe, although most relevant references were published within the past decade, particularly in the past five years. We also consulted reference lists of identified articles and of applicable reports. We did not limit our search to papers published only in English. For each article reviewed, we reviewed references in that article to identify other relevant publications that might have been missed by the PubMed search, and repeated this process for the additional relevant articles reviewed. Literature searches were conducted primarily in April–June and November–December 2024. The final reference list was generated on the basis of relevance to the scope of this article.

## Introduction

Hypertension is the leading risk factor for early death worldwide, killing more people than any other condition and more than all infectious diseases combined.^1^ Approximately 80% of these deaths occur in low- and middle-income countries (LMICs). If uncontrolled, high blood pressure greatly increases the risk of ischaemic heart disease, stroke, other cardiovascular diseases, chronic kidney disease, and dementia.^2^ Hypertension and its complications have enormous economic costs for patients and their families, health systems, and national economies from direct medical expenses as well as lost wages and productivity.^1^ Deaths from hypertension increased from an estimated 6.7 million in 1990 to 10.8 million in 2019, and, without urgent action, are projected to increase to more than 14 million by 2050.^3^

Prevention of hypertension, for example through replacement of regular salt with potassium-enriched low-sodium salt, is possible, but even if successful would leave more than a billion people in need of antihypertensive treatment.^4^^,^^5^ Improved treatment could save more adult lives than any other adult health care intervention, could avert the projected increase in deaths despite the increasing and aging population, and is essential to achieve the Sustainable Development Goal 3.4 of a one third reduction in the rate of premature mortality from non-communicable diseases by 2030.^6^^,^^7^

## Scaling up hypertension treatment

First-line oral antihypertensive medications are safe, effective, and available in generic form; some countries and some health care systems provide effective treatment to a high proportion of their patients, demonstrating that progress is possible and that the result is a substantial reduction in cardiovascular deaths and an increase in healthy life expectancy.^8^ Only approximately 1 in 5 of the estimated 1.3 billion adults with hypertension worldwide, and less than 10% in LMICs, have blood pressure controlled to 140/90 or below.^1^ Improving hypertension control will depend, fundamentally, on changes in the structure of health care that increase access and reduce barriers to evidence-based, patient-centered treatment.

The asymptomatic nature of hypertension makes it the ‘silent killer,’ inapparent to patients, who are less likely to continue in care and less willing to pay for treatment than for symptomatic albeit less deadly conditions. The first symptom of hypertension is often a heart attack or stroke. Hypertension treatment programs depend on accessible basic primary health care services, including access to essential antihypertensive medicines. As these systems are suboptimal in much of the world, progress improving hypertension control has been slow, even in higher-income countries and in countries with strong primary health care systems.^1^

The World Health Organization (WHO) established the HEARTS technical package to improve hypertension treatment in primary care settings. HEARTS consists of five components: a standard hypertension treatment regimen, uninterrupted supply of quality-assured drugs and blood pressure monitors, team-based care, patient-centered services, and an effective information system.^1^ The single most important indicator to measure progress—although one not tracked systematically in many health systems—is the community control rate, i.e., the proportion of all people in an area with hypertension who reach the blood pressure goal. Increasing the control rate to 50% globally would prevent between 76 and 130 million deaths over 35 years.^3^

Each component of the WHO HEARTS strategy improves hypertension control.^9^ Simple medication regimens, once-a-day dosing, and single pill fixed-dose combination medications improve blood pressure control.^10^ Dispensing multi-month (e.g., 3- or 6-month) prescriptions to clinically stable patients may increase adherence over commonly used (e.g., one-month) prescription durations.^11^ Patient education, particularly when individualized and reinforced at every clinical visit, can improve medication adherence and blood pressure control. Use of digital tools, including mobile applications for blood pressure and medication tracking, text messaging and other patient reminders, and self-management aids are promising methods to augment other educational and behavioral interventions.^12^ Information systems that enable health care workers and program managers to determine what proportion of patients have their blood pressure under control and easily reach out to those who have interrupted treatment or whose blood pressure needs additional attention can substantially increase control rates.^13^

## Barriers to patient antihypertensive medication adherence

Increasing the proportion of patients who continue taking medications and have their blood pressure controlled is the central challenge of hypertension treatment programs. Globally, hundreds of millions of people are aware of their hypertension diagnosis but do not have their blood pressure controlled. This reality emphasizes the importance of better treatment programs and the inefficiency and limited impact of community screening for hypertension.

The health system, not patient adherence, is the strongest predictor of continued treatment. Strong health care systems can increase the proportion of patients with blood pressure controlled to 90%.^14^ Globally, problems include stockouts of medications, inadequate primary health care infrastructure, lack of a team-based approach to care, inconvenient hours and locations of clinical and pharmacy services, services that do not make patients feel respected, and ineffective information systems that do not allow providers to know when patients are out of care or facilitate their return to services.^15^ Each of these problems needs to be addressed to implement the HEARTS strategy successfully.

One barrier to treatment continuation merits particular attention, both because it is so important and because there are important misconceptions about it: The requirement in some primary health care systems that patients pay for anti-hypertensive medications. Patients are less likely to continue in care, take medications, and have their blood pressure controlled if they must pay for medications.^16^ Although a single patient's medicines can cost less as little as US$5 per year, costs in many systems are much higher, and many patients are unable to afford medications in the face of poverty and the need to pay for essentials such as food and housing. User fees, particularly those that require payment at the time of service, reduce patient access to health care and are a regressive form of health care financing.^17^^,^^18^ Removing user fees has a large potential benefit, particularly in LMICs and for low-income populations, and increases uptake of and adherence to a wide range of health services.^19^

Table S1 (Supplementary Material) summarizes published articles that demonstrate the benefit of reducing or eliminating patient out-of-pocket medication costs; it is not a compendium of all available evidence. Although most studies are from high-income countries, evidence from hundreds of thousands of patients consistently demonstrates large decreases in adherence—and increases in cardiovascular events—from even modest increases (e.g., $5 or $10 dollars per prescription in high-income countries) in costs. It is therefore a near-certainty that similarly large decreases in adherence are occurring because of medication fees in LMICs, although these are less well documented in the published literature.

## Provision of free antihypertensive medications

There appear to be two broad reasons health systems require that patients pay for antihypertensive medications: a conceptual error and a lack of resources. The first is the misperception, apparently common in some parts of the world, that patients will not value health care services unless they pay at least some of the costs. The reality is quite different: Studies consistently document that copayments at the time of service reduce adherence, increase health inequity, can lead to medication underuse (e.g., partial or skipped doses), and increase uncontrolled hypertension.^19^, ^20^, ^21^, ^22^ Free blood pressure medicines reduce missed visits.^23^ In China, free antihypertensive medications resulted in reduced stroke mortality.^24^ In Ghana, patients receiving free medications were more than twice as likely to achieve blood pressure control as those paying out-of-pocket.^25^

The benefits of providing medications free of charge go beyond blood pressure control and other medical outcomes. In Canada, providing medicines at no charge to people with conditions such as hypertension and diabetes improved health measures related to these conditions, increased patient perceptions of the quality of their care, and appeared to improve patients' ability to cover costs for other basic necessities.^26^ A follow-up analysis showed that eliminating out-of-pocket medication costs reduced total health spending, with most savings from reduced hospitalizations.^27^

Hypertension control correlates with individual and country income. Lower-income people in LMICs have the least access to care and will benefit the most from policies such as free medications.^28^ A study in China showed that vulnerable populations with lower income or educational level, rural residence, and age 65 years and above were more likely to have improved access to antihypertensive medications when available at no charge.^29^ Additional trials that document the health and economic benefits of eliminating patient out-of-pocket costs for antihypertensive medications, as well as the impact on promoting equitable access and reducing health disparities, can add to the growing evidence base.

Provision of free medications has been effective at improving adherence and outcomes in other programs. Free care of HIV, tuberculosis, and malaria has been essential to the global scale-up of treatment programs. Free vaccines are a core component of child survival initiatives.^30^ Free care has also been shown to be effective in combatting other non-communicable diseases. Cost-free nicotine replacement medications or bupropion increased smoking quit rates.^31^ Free asthma medications reduced hospital admissions by one third.^32^

The second reason for the requirement that patients pay for medications is the need to fund health care services. Despite the availability of generic antihypertensive medications costing as little as US$5–10 per patient annually, the high prevalence of hypertension (20–40% of adults in most countries) results in substantial aggregate costs, often exceeding current budgets for medication provision through primary health care services.^33^ Since health care in general, and primary health care in particular, is substantially underfunded in many countries, many health systems may at present lack adequate resources to provide medications free of charge to patients.

Increased adherence reduces health complications from cardiovascular diseases and other conditions and, in many contexts, will save more money from prevented hospitalizations, strokes, and heart attacks than would have been generated from patient copayments.^34^^,^^35^ However, unless payment models redirect savings from prevented hospitalizations into increased funding for primary care services, these budgetary savings will not translate into additional resources for medications and other care of people with hypertension.

Making essential antihypertensive medicines available and accessible in many LMICs may require increased government funding that can be sustained over time. In an area in which 40% of people are over age 30 and 30% have hypertension (approximate global averages for LMICs), 50% are treated effectively (an ambitious global target), and the per capita cost for medications is $5/year, this would require a yearly expenditure of only US$0.30 (30 US cents) per capita.

There is a global commitment to provide access to services without financial hardship.^36^ For countries in which patients pay health insurance premiums, ensuring that these payments are income-sensitive and that there are no payments requested at the time of clinical service or medication dispensing can improve health care access and outcomes. For lower-income households, many of which do not participate in formal health insurance schemes, voluntary community-based health insurance programs, in which community members pool funds to pay health care costs, can improve health care utilization, although premium costs and copayments are barriers to access.^37^

Optimal health care financing mechanisms use prepayment methods such as taxation and insurance premiums rather than direct patient payments at the point of service delivery. This approach facilitates the pooling of funds across the population, distributes financial risk, and increases equity and sustainability.^38^ To support lower-income countries, time-slice funding, whereby countries commit to a steady increase in the proportion of funds paid from their national budgets (which for LMICs could be over a time horizon of 10 years or longer) with catalytic funds provided by donors or financial institutions could increase both access and funding stability.

## Implementation considerations

Removing patient out-of-pocket medication payments requires careful attention to the details of implementation to avoid unintended consequences. It is critical to ensure adequate drug supply. In Uganda, 23% of public health unit facility managers reported inadequate drug availability after removal of user fees, despite additional government funding earmarked for medication purchases.^39^ In Zambia, nearly 20% of patients stated that drug availability at public health centers had worsened after user fees were abolished.^40^ In many facilities, user fees provide a large proportion of the flexible funding needed for facility operations or provider pay.^41^ Removal of payment for medications and dispensing requires alternative sources of funding, for example taxation or health insurance.

Standardization of treatment protocols with three core medications creates economies of scale and substantial reductions in unit costs, increasing the number of patients who can be treated with the same budgetary investment. Strategic procurement and distribution strategies, including supply chain optimization, can reduce costs. Similar savings can be achieved from selecting a single statin agent; many people with hypertension will gain a substantial additional reduction in morbidity and mortality from co-prescription of a statin.^42^ Antihypertensive medications and statins provide a substantial proportion of the potential health benefits of primary health care for adults.

Provision of free medications is not a panacea. In Thailand, where all patients have access to free antihypertensive medications, control rates remain low due in part to limited clinic operational hours.^43^ In Nepal, one hospital-based study found low medication adherence despite free medications, possibly because of the inconvenience of hospital-based services and limited awareness of the need for continued treatment.^44^ Adequate drug supplies and sufficient clinical staff are essential to manage increased demand and utilization.^45^

Decentralization of services to facilities closer to patients' homes, as done in the treatment program run by the Government of India, allows for more patient-centered services, reducing the distance barrier for patients and making the provision of free medication even more effective in improving blood pressure control rates. Services in the community were associated with higher control rates even though care was free at all levels of the health care system.^46^ In Nigeria, low-cost, fixed dose combination (FDC) blood pressure medications were included in the country's Essential Medicines List to align with national hypertension treatment guidelines and standard treatment protocol. This policy could, if there are adequate budgetary resources to procure the medications, reduce out-of-pocket costs for antihypertensive medications for patients, especially those enrolled in health insurance programs.^47^ However, inclusion of FDCs can increase per-patient costs for government and reduce antihypertension drug supply if low-cost FDCs are not available.

## Conclusion

The WHO HEARTS technical package provides a comprehensive approach to improve hypertension treatment in primary care settings. An important but underutilized component of hypertension management is the provision of antihypertensive medications at no cost to patients. This increases adherence, improves blood pressure control, reduces cardiovascular events and health care costs, and may increase the ability of patients to meet other basic needs, including food and housing.

Providing medications at no cost to patients, combined with other elements of the HEARTS package, can strengthen primary health care, advance universal health coverage, and promote progressive universalism in primary care. Focusing on hypertension—a condition highly amenable to treatment—can bridge the gap between current practice and optimal care, averting millions of premature deaths and establishing a model to address other chronic and non-communicable diseases.

## Contributors

TRF led the drafting and revision of the manuscript. RG and BB reviewed and edited the manuscript, conducted literature review, and contributed to overall revision. SC, NO, DD, and VT reviewed and edited the manuscript and provided specific input to particular geographic and technical areas. All authors accept responsibility for the decision to submit for publication.

## Declaration of interests

We declare no competing interests.
