# Supplementary material for: Unlocking health equity by eliminating copayments for essential antihypertensive medications
Source: eClinicalMedicine. 2025 Jan 30;81:103094. doi: 10.1016/j.eclinm.2025.103094 (PMC11833357; doi:10.1016/j.eclinm.2025.103094)
Supplement: Supplementary Table S1 [file mmc1.docx]

**Table 1. Summary of included studies and relevant characteristics (*n* = 31).**

| **Author and year** | **Country and income class** | **Sample included** | **Relevant findings** |
| --- | --- | --- | --- |
| Rezayatmand R (2013) | Literature review | 47 relevant publications | Out-of-pocket payments create a financial barrier and can decrease use of preventive services and uptake of preventive medications. |
| * Ellis JE (2004) | USA (high) | 2,258 secondary and 2,544 primary prevention non-Medicaid patients | Medication adherence to statins was 3.2 times higher with copayments of US$10 than US$20 or higher (odds ratio 3.23) |
| * Ye X (2007) | USA (high) | 13,765 patients with complete copayment information | Medication adherence to statins after coronary heart disease hospitalization was 38% lower with copayments of US$20 or higher than of US$10 (odds ratio 0.62) |
| * Gibson TB (2006) | USA (high) | 142,341 new statin users and 92,344 continuing statin users | A $10 increase in statin cost sharing was associated with an 8.9% decrease in the odds of being adherent for new users and an 11.9% decrease for continuing users |
| * Gibson TB (2006) | USA (high) | 24,113 new statin users and 93,253 continuing statin users | A 100% copayment increase reduced monthly adherence by 2.6% and 1.1 % among new and continuing users, respectively |
| * Goldman, DP (2006) | USA (high) | 62,274 patients starting cholesterol lowering drugs | A 100% copayment increase (from US$10 to US$20) decreased the fraction of fully compliant patients by 6 to 10%, depending on patient risk |
| * Federman AD (2001) | USA (high) | 1,908 Medicare beneficiaries with a history of CHD or myocardial infarction | Significantly lower statin use among patients who lack drug coverage (4.1%) than those with employer-sponsored drug coverage (27.4%) |
| * Chernew ME (2008) | USA (high) | 108,126 employees and dependents enrolled in company health care plans | Decreasing copayment from $5 to zero for generic medications and by 50% for brand-name medications reduced nonadherence by 7-14% for four NCD drug classifications |
| * Landsman PB (2005) | USA (high) | 1,630,000 enrollees in managed health care plans | Increasing copayments increased medication discontinuation rates from 2 to 5.5 times over control group for four NCD drug classifications |
| * Huskamp HA (2003) | USA (high) | 150,222 enrollees in employer health care plans | Increasing copayments increased ACE and statin discontinuation rates 2.5 and 1.9 times over comparison group, respectively |
| * Cole JA (2006) | USA (high) | 12,776 patients with chronic heart failure | A $10 increase in ACE drug copayments was associated with a 2.6% decrease in medication possession ratio and a predicted 6.1% increase in risk of CHF hospitalization |
| * Taira DA (2006) | USA (high) | 114,232 managed care patients with hypertension | Relative to antihypertensive medications with a $5 copayment, compliance was 24% lower with drugs having a $20 copayment |
| Watson SI (2016) | Malawi (low) | 13 health centers in Meno District, Malawi (8 public, 1 private, 4 nonprofit) | Introduction of user fees was associated with a 68% decrease in health center outpatient attendance; removal of user fees was associated with an 352% increase in attendance |
| Maina T (2015) | Kenya (lower-middle) | 250 primary care facilities in Kenya | Removal of user fees increased total outpatient services (visits and re-attendance) delivered in public facilities by 25% for children five years and under and 37% for the total population over age five |
| Qin VM (2020) | Systematic review | 17 studies from 12 LMICs (5 upper-middle, 5 lower-middle, 2 low) | Reduced user charges were associated with improved health outcomes, particularly for lower-income groups and children in LMICs |
| † Beuermann D (2016) | Jamaica (upper-middle) | 35,434 national survey respondents | Free universal public health care with no user fees resulted in a 34% decrease in lost work days due to illness |
| † Sosa-Rubí SG (2016) | Mexico (upper-middle) | 1,491 adults with diabetes | Health care provision with no patient co-pays resulted in a 20.3% higher likelihood of appropriate HbA1c glucose control |
| † Huang F (2017) | China (upper-middle) | 1,764 urban-dwelling survey respondents | Increasing patient out-of-pocket costs for health care services by 2-3 times decreased the probability of medical service utilization by approximately 9 percentage points |
| Maciejewski ML (2010) | USA (high) | 7,090 hypertensive patients at VA medical centers | A $5 copayment increase (from $2 to $7) decreased antihypertensive medication adherence by 3.2% |
| Kostova D (2017) | USA (high) | 9,000 low-income adults in 18 states | Introduction of drug copayments was associated with an average 7.7 percentage point increase in uncontrolled hypertension |
| Attaei MW (2017) | 20 countries representing all income levels | 158,247 hypertensive patients | Patients able to afford antihypertensive drugs were 42% more likely to use medicine and 13% more likely to have controlled blood pressure (than those unable to afford medicine |
| Zhou Q (2023) | China (upper-middle) | All stroke deaths in Deqing, Zhejiang province | Provision of free antihypertensive medications resulted a 10% reduction in stroke mortality |
| Harrison MA (2021) | Ghana (lower-middle) | 310 hypertensive patients | Obtaining antihypertensive medicines at no cost through national health insurance was 2.2 times more likely to result in BP control than paying out-of-pocket |
| Persaud N (2020) | Canada (high) | 786 primary care patients | Free distribution of essential medicines vs usual access resulted in 11.6% greater adherence and a reduction in systolic BP pressure of 7.2 mm Hg |
| Stein DT (2024) | 44 countries (low and middle) | 110,000 people living with hypertension | Eliminating socioeconomic-based disparities in hypertension diagnosis and treatment would reduce CVD case rates 1.7 times more among the lowest vs highest quintile income groups |
| Guo Z (2021) | China (upper-middle) | 340,638 patients with hypertension and diabetes | Availability of medications at no cost to patients improves access, especially among vulnerable populations |
| Lindson N (2021) | Countries on 5 continents representing all income levels | 112,159 patients receiving smoking cessation medicines in primary care | Providing cost-free smoking cessation medications increased smoking quit rates in primary care by 36% |
| Comaru T (2016) | Brazil (upper-middle) | All public hospital admissions of asthma patients age 1-49 nationwide | Provision of free medicines to treat asthma reduced asthma-related hospital admissions by 33% |
| Choudhry NK (2011) | USA (high) | 5,855 patients post- myocardial infarction | Coverage of prescriptions with no cost to patients reduced rates of subsequent stroke by 31% and of vascular events by 14%, with an 11% reduction in total and CVD-specific health care costs |
| Dragomir A (2010) | Canada (high) | 59,647 patients with hypertension | Patients with low medication adherence were more likely to have coronary heart disease (7%), cerebrovascular disease (13%), and chronic heart failure (42%) over 3-year follow-up, with average 37% increase in total per-person health care costs |
| Eze P (2023) | 20 countries (low and middle) | 1,012,542 persons in 221,568 households | Insured households had 60% higher health care utilization and 58% higher use of outpatient health services, with 6% lower out-of-pocket health expenditure and 31% lower likelihood of catastrophic health expenditure; increasing adherence would provide a net economic gain |

* Included in Rezayatmand (2013) analysis.

† Included in Qin (2020) analysis.
